# Interexaminer Reliability and Validity of Quantity of Cervical Mobility during Online Dynamic Inspection

**DOI:** 10.3390/diagnostics12020546

**Published:** 2022-02-21

**Authors:** Leire Leonet-Tijero, Jaime Corral-de-Toro, Jacobo Rodríguez-Sanz, Mar Hernández-Secorún, Hugo Abenia-Benedí, María Orosia Lucha-López, Sofía Monti-Ballano, Julián Müller-Thyssen-Uriarte, Héctor Tricás-Vidal, César Hidalgo-García, José Miguel Tricás-Moreno

**Affiliations:** 1 Faculty of Health Sciences, University of Zaragoza, C/Domingo Miral s/n, 50009 Zaragoza, Spain; leirelt17@gmail.com (L.L.-T.); jaimecorral.fisio@gmail.com (J.C.-d.-T.); marhsecorun@unizar.es (M.H.-S.); hugoabenia1@gmail.com (H.A.-B.); smonti1395@gmail.com (S.M.-B.); julianmuller.jmt@gmail.com (J.M.-T.-U.); jmtricas@unizar.es (J.M.T.-M.); 2 Physiotherapy Research Unit, University of Zaragoza, C/Domingo Miral s/n, 50009 Zaragoza, Spain; hjtricas@gmail.com; 3 Faculty of Medicine and Health Sciences, Universitat Internacional de Catalunya, Sant Cugat del Vallès, 08195 Barcelona, Spain; jrodriguezs@uic.es; 4 ACTIUM Functional Anatomy Group, 08028 Barcelona, Spain

**Keywords:** neck, physical examination, telemedicine

## Abstract

Background: Physical therapists routinely measure range of motion (ROM) of cervical spine. The reliability of the cervical range of motion (CROM) device has been demonstrated in several studies, but current evidence on the validity and reliability of the visual inspection is contradictory. The aim is to assess the validity and interexaminer reliability of the online visual inspection of active cervical ROM in physiotherapy students. Methods: Flexion, extension, both lateral flexions and rotations of a single participant were measured using CROM. Online visual inspection of 18 physiotherapy students against CROM was registered. Results: The validity, against CROM, of the online visual inspection of the active ROM ranged from good to excellent (Intraclass Correlation Coefficient (ICC) 0.83–0.97). Interexaminer reliability of the online visual inspection had favorable outcomes in all cervical movements in the three physiotherapy courses (ICC 0.70–0.96), with the visual inspection of the rotations being the most reliable (ICC 0.93–0.97). Interexaminer reliability of the classification of mobility was poor to good (Kappa 0.03–0.90). Conclusions: The interexaminer reliability and validity of the quantification of active cervical movement during online visual inspection was shown to be good to excellent for flexion-extension and lateral flexions and excellent for rotations.

## 1. Introduction

During the physical examination of the cervical spine, physiotherapists routinely assess the range of movement (ROM) [1,2]. Cervical ROM examination is a basic component for patients with cervical pain [3,4] and the need for a quantification of the functional loss has been raised [5].

The physical examination consists of asking the patient to perform the basic kinesiological movements, including the active ROM for the flexion, extension, both lateral flexions and rotations [2,6] in a sitting position [6]. According to Magee et al. [7], tests of active range of movement (AROM) are used to identify or determinate limitation on motion, patient’s willingness to move, and to identify the ROM in which the patient reports symptoms. Many non-radiological instruments have been developed in recent years, including goniometry, inclinometry [8], electrogoniometry, electromagnetic, ultrasonic and optoelectronic systems [9] and smartphone apps [10,11,12], those being a cheaper and more accessible option for physiotherapists.

Additionally, physical therapists can measure a patient’s ROM using “Cervical Range of Motion device” (CROM) [13]. In recent years, several studies have been developed to determine normal ranges of cervical ROM using this device [5,14]. All cervical spine movements can be measured without manual adjustment, eliminating a deficient manual technique possibility [15], and this can be managed by a single evaluator [16] with the patient seated [17]. Moreover, it does not require advanced calculations that would require loss of extra time [16] and it measures flexion, extension, lateral flexions and rotations through a compass-like instrument with a magnetic booster [13]. It has been suggested that with an experienced professional in the CROM use and with a standardized method, cervical active ROM is reproducible enough in a single session for patients with or without cervical pain [18]. Studies have demonstrated CROM reliability measuring cervical movements in the three movement planes, for both healthy [17,19] and symptomatic subjects [13,20]. Furthermore, it was found in several studies that the intra- and interexaminer reliability in the evaluation of active cervical ROM was moderately higher when the subjects were measured through the CROM device [15,21,22].

Another tool used for the cervical spine study is the visual estimation (VE) or visual inspection [13], which aims to document visible defects, general positioning and posture, functional deficits or lesions detectable by the eye with or without the assistance of devices. Recent evidence is contradictory about the measurement of cervical ROM through visual inspection [23] and the need for protocols and tools for clinical assessment [24], and to investigate the ROM, measurement techniques for different joints have been suggested [25]. Some studies declare that visual inspection may be the least reliable and valuable method for cervical ROM evaluation [1,17,23,26], while others conclude that cervical active ROM can be measured by visual inspection, being just as reliable as a measurement tool and with a moderate to significant interexaminer reliability [27,28]. Another study found intra- and interexaminer reliability ranges from significant to perfect for the ROM estimation through visual estimation [23].

It is known that cervical spine examination can not only be carried out in face-to-face interviews, but also that the visualization of videos can be a possible new way of observing patients [29,30]. Thus, “telemedicine” has been reported as a useful means for professionals who run many physical exploration components [30] to provide remote rehabilitation services [31] for people who are physically or geographically isolated from the rehabilitation specialists [32,33,34]. It can also be useful for health emergency situations, such as the one currently experienced due to the COVID-19 pandemic [35]. Moreover, some reviews demonstrated that the objective physiotherapy assessment of musculoskeletal injuries using telemedicine was technically feasible, since validity and reliability ranged from good to excellent [31,34], and a previous study analyzing telerehabilitation on chronic low back pain showed an accurate interexaminer reliability of ROM and orthopedic test [36].

The main aim of the current study was to analyze the interexaminer reliability and validity of online visual inspection of the quantity of active cervical movement. The secondary aims were to analyze the interexaminer reliability of a classification of cervical mobility and to collate the difficulties and potential improvements while analyzing online visual estimation of cervical ROM.

## 2. Materials and Methods

### 2.1. Study Type and Sample

The following is a descriptive validity and reliability study. This validity and reliability study was carried out at the University of Zaragoza (Spain). The study was approved by the local ethics committee “Comité Ético de Investigación Clínica de Aragón” (PI21/432). One asymptomatic physiotherapist was repeatedly filmed performing different cervical motions with different ROM and in the three cardinal planes. This physiotherapist was informed about the procedure and signed the informed consent form.

Inclusion criteria for the filmed subject were: (1) being a physiotherapist who is over 18 years of age; and (2) being able to simulate different ranges of movement of the cervical spine in all cardinal planes of movement.

An exclusion criterion for the filmed subject was the presence of any contraindication for movement in the cervical spine [1,9,37,38].

Inclusion criteria for the examiners were: (1) to be students in the second, third, or fourth year/course of the Degree of Physiotherapy (PT) [39]. The students in the first course were excluded as they had not taken the course “Kinesiology” yet. Of all volunteers, six from each course were randomly chosen. Thus, 18 physiotherapy students took part as examiners of the videos.

**Figure 1:**
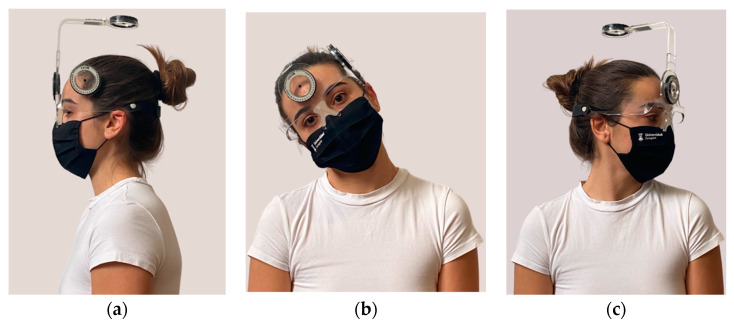
Position of CROM device for: (**a**) Sagittal plane for flexion-extension movement; (**b**) Frontal plane for lateral flexion movement; (**c**) Transversal plane for rotation movement.

The subject was sitting in a 0.45-meter-high backless chair, with the knees bent at 90°, looking ahead, the arms were hanging and the feet were flat on the floor. The CROM device (Floating compass; Plastimo Airguide, Inc., Buffalo Groove, IL, USA) was positioned on the subject´s head to measure the movements in the sagittal and frontal plane with an inclinometer and in the transversal plane with a compass stabilized by a magnetic equipment installed around the subject´s neck (Figure 1).

The subject was instructed to perform 8 movements with different ROM in each one of the cardinal planes of the cervical spine (flexion, extension, both lateral flexions and rotations) [1,9,18]. The final ROM of every 8 videos in each of the three cardinal planes (flexion–extension, right–left lateral flexions and right–left rotations) were registered by another physiotherapist with the CROM device. The final ROM of each video in each of the three cardinal planes (flexion–extension, right–left lateral flexions and right–left rotations) was registered by another physiotherapist with the CROM device. This register constituted the “gold standard” for the validity study and the variable was denominated “ROM of CROM device”. Various studies have achieved a moderate to excellent reliability and validity of the CROM to measure active cervical movement [14,17,22,40]. All movements were recorded with a Huawei P20 phone that was placed on a tripod at a height of 1.05 m and at a distance of 1 m from the subject [18].

Finally, twenty-four videos were recorded, eight videos for each plane of movement. Videos were included to be analyzed in three questionnaires created in the Google Forms platform, with questions related to the degrees of movement in visual inspection and the qualitative classification of normal or impaired mobility (hypomobility or hypermobility) observed in the different videos. One questionnaire was created for the 8 videos of flexion-extension, one for the 8 videos of the lateral flexion, and a third one for the 8 videos of rotation. Among the 18 examiners, 6 answered the flexion–extension questionnaire (2 examiners from the second course, 2 examiners from the third course and 2 examiners from the fourth course), 6 answered lateral flexions and 6 answered rotations, with the same distribution [39].

### 2.2. Study Outcomes

The examiners were asked to perform a visual inspection of the 8 videos of the corresponding cervical movement while watching each video a maximum of three times. The questionnaires were answered anonymously, and they gathered data on participants’ gender and physiotherapy course, defined as sociodemographic variables.

The examiner answered four questions related to each video and completed the questionnaire after answering the questions about the 8 videos (4 questions for each video, 2 questions for each of the senses of movement for each video).

The first question was related to the ROM in visual inspection (quantitative variable) in cervical active ROM observed for each movement in the analyzed cardinal plane. Examiners would answer with an absolute angular value for the flexion as well as for the extension, following the same procedure in both lateral flexions and both rotations. This variable was registered as “ROM in visual inspection” for each movement.

The second question was related to the classification of mobility (qualitative variable), in cervical active ROM observed for each movement in the analyzed cardinal plane. Examiners would judge if the watched motion was hypomobile, normal or hypermobile [27,41]. This variable was registered as “classification of mobility” for each movement.

Furthermore, two questions were added at the end of the questionnaire for the students to evaluate their participation subjectively [42], expressing their difficulties in determining the ROM, and suggesting new ideas for the videos in the online measurement.

### 2.3. Statistical Analysis

The software IBM SPSS Statistics 21.0 for Windows was used [43]. Descriptive statistics were calculated for the examiners’ sample, analyzing frequencies and percentages for gender, as a qualitative variable, and relating them with the qualitative variable named “physiotherapy course” of the examiners.

Interexaminer reliability of the quantitative variable named “ROM in visual inspection” was analyzed calculating the Intraclass Correlation Coefficient (ICC) with a confidence interval of 95% [19,44]. The estimation of the ICC was based on the two-factor random effects model, in which both the effects of individuals and the effects of the measures were random and the “consistency” agreement between examiners was chosen [18].

Interexaminer reliability of the qualitative variable named “classification of mobility” was analyzed calculating the Kappa index with a confidence interval of 95% [45].

ICC was calculated to measure validity of online visual inspection for the quantitative variable named “ROM in visual inspection” in comparison to the gold standard, “ROM of CROM device”. The estimation of the ICC was based on the two-factor random effects model, in which both the effects of individuals and the effects of the measures were random and the “consistency” agreement between examiners was chosen [46].

ICC or Kappa value (from 0 to 1) for validity and reliability was interpreted according to the following classification: poor <0.5; moderate <0.5 to 0.75; good 0.75 to 0.90; and excellent >0.90 [9,46].

## 3. Results

### 3.1. Sociodemographic Variables

Of the 18 examiners, 44% were men and 56% were women. Regarding students’ gender according to physiotherapy course, three women and three men from the third and fourth courses and four women and two men from the second course participated.

### 3.2. Reliability and Validity Studies

#### 3.2.1. Interexaminer Reliability of the “ROM in Visual Inspection”

The interexaminer reliability results related to degrees of movement showed good to excellent value for flexion–extension (ICC 0.75–0.96) and lateral flexions (ICC 0.81–0.93), and excellent value for rotations (ICC 0.93–0.97) for each physiotherapy grade. ICC with the lower bound and upper bound of the 95% confidence interval are listed in Table 1.

#### 3.2.2. Interexaminer Reliability of the “Classification of Mobility”

The interexaminer reliability results related to classification of mobility showed poor to moderate value for flexion-extension (Kappa 0.03–0.62) and lateral flexions (Kappa 0.26–0.54), and moderate to good values for rotations (Kappa 0.58–0.90) for each physio-therapy grade. Kappa index values are shown in Table 1.

#### 3.2.3. Validity of “ROM in Visual Inspection”

The validity of “ROM in visual inspection” when compared to CROM device demonstrated good to excellent values in all movements, with ICC values from 0.83 to 0.96 (Table 2).

### 3.3. Examiners Difficulties and Advice

**Figure 2:**
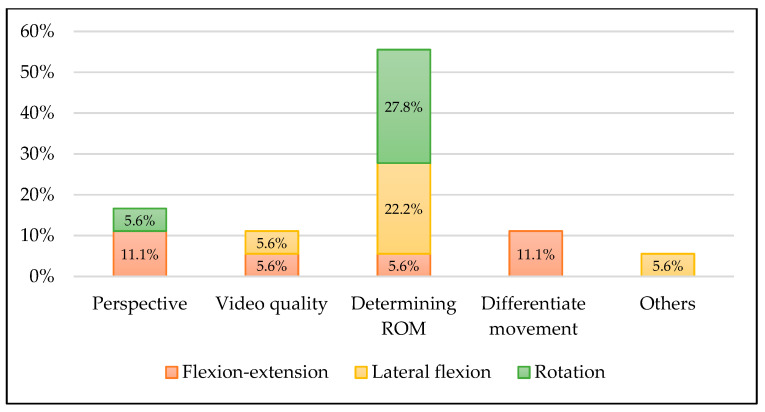
Difficulties of range of motion estimation described by the examiners.

Figure 2 shows the examiners’ answers to the question regarding difficulties in measuring cervical ROM. Determining lateral flexions and rotations ROMs was the most difficult issue for the students.

**Figure 3:**
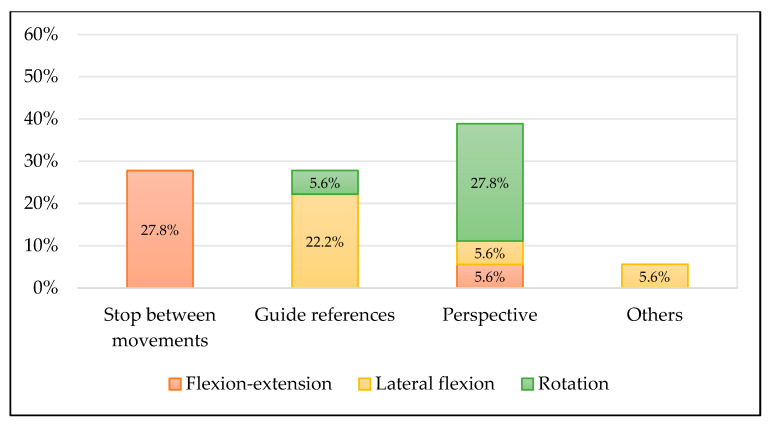
Potential improvements proposed by the examiners.

Figure 3 shows the potential improvements for the recording methodology of cervical ROM that examiners suggested. Stopping or dividing the movement in the sagittal plane in two different flexion and extension sequences and a change of perspective in transversal plane movements for the video capturing were the main proposed solutions by the examiners.

## 4. Discussion

This study examined the validity, compared with a gold standard (CROM device), and the interexaminer reliability of online visual inspection of the quantity (ROM and classification of mobility) of active cervical movements.

The results indicated that the interexaminer reliability ranged from good to excellent for ROM in visual inspection and the interexaminer reliability of classification of mobility was poor to good. The validity of online visual inspection of the ROM of the active cervical movement ranged from good to excellent.

Contradictory findings can be found in the literature regarding the reliability of visual estimation of the cervical movement. According to Nordin et al. [2], interexaminer reliability (Kappa 0.96–1.00) increased as the level of disease prevalence decreased, in healthy controls of cervical spine. Whitcroft et al. [27] found in their study good to excellent intra- and interexaminer reliability (ICC 0.75–0.99) for cervical active ROM, and Hoppenbrouwers et al. [39] found moderate reproducibility in active and passive cervical examination, in which early graduated physiotherapists participated. However, other studies suggest that visual inspection of the cervical spine is not consistent for flexion, extension and lateral flexions [23] and according to Pool et al. [46], interexaminer reproducibility of cervical spine physical exploration is not reliable, except when following a standardized protocol. Youdas et al. [21] also studied visual inspection reliability to evaluate the cervical spine and in spite of following defined protocols, their study obtained from poor to moderate reliability.

Our study showed an interexaminer reliability of online visual inspection of cervical active ROM from good to excellent in flexion and extension movements for second- and fourth-course PT students and from good to excellent reliability for third-course PT students. According to lateral flexions exploration, many studies [21,23,24,39] conclude that cervical range of movement measurement is not reliable, although our study has shown moderate to excellent interexaminer reliability, with third-course students being the most reliable.

In relation to the variable “classification of mobility”, our study showed poor to moderate interrater reliability for flexion–extension and lateral flexions and from moderate to good in rotation. The level of PT course did not seem to influence the degree of reliability. Vikari et al. [24] determined concrete angular values to classify a movement as hyper/hypomobile or normal obtaining from poor to moderate reliability (Kappa 0.41–0.56). However, Hoppenbrouwers et al. [39] classified extension as restricted if the line between the nose and forehead did not reach horizontality. For lateral flexions and rotations, measurements were taken from tragus to acromion and from chin to acromion, obtaining moderate interexaminer reliability (Kappa 0.53). All these studies classified mobility according to a range of degrees of movement, while, in our study, we did not provide any standardized amount of degrees of ROM to classify a normal or an impaired motion. It is possible that providing a proper training guided by determined standardized ROM in the different planes, according to the age of the videotaped subject, as promoted by Swinkels et al. [1], would improve interrater reliability.

It is considered that cervical ROM should be easily estimated via video during a videocall [47]. However, there is an absence of validity studies for visual estimation of the cervical ROM via video [48]. Our study analyzed the validity of online visual inspection of the active ROM compared with the CROM. The CROM device has been considered as a reference standard for measuring cervical ROM [18,49,50,51]. Whitcroft et al. [27] ran a similar study, obtaining visual feedback through photos, where they obtained moderate validity. Instead, our study obtained from good to excellent validity in all planes of movement, similarly to Allahyari et al. [52], finding a good to excellent validity between a Kinect sensor and an electrogoniometer. Both Kinect and video recording require that the tracked region face the sensor or be visible to the camera. Christensen et al. [48] achieved greater accuracy with a 2D video analysis via software than visual estimation for lateral flexion and right rotation in infants. However, their visual estimation of the ROM was consistent and the time and technology necessary for the evaluation was lower than with video analysis via software.

Nevertheless, in their systematic review, Williams et al. [17] classified visual inspection as the least reliable method to assess cervical ROM. However, although it is necessary to investigate visual inspection validity with larger samples and symptomatic subjects, statistically significant results were obtained in our study and validity was provided through the standardization of the recording methodology [21,24,27,46,51].

On the other hand, the singularity of our study is that visual inspection of the cervical spine is performed via online. The current implication of technology in physiotherapy has driven investigators to compare visual inspection with bidimensional analysis [48], photography [27], photogrammetry [53] or 3D systems of movement analysis obtaining a considerable reliability [9,37,38,46], although their clinical utility becomes challenged as they require complicated and expensive measurement procedures [9,54]. In contrast, our study has demonstrated the possibility of using online visual inspection for determining the quantity of movement in a reliable way without having a complicated and expensive measurement protocol.

Likewise, online visual inspection focused on the ROM measurement could be used as a tool in telemedicine [26,30,31,36,55]. Until now, telerehabilitation utility has been studied among different body regions as lumbar spine, ankle, elbow or knee [30,31,34,36,56,57], obtaining favorable reliability for ROM [35,58]. Our study could provide a new line of research focused specifically on visual inspection of cervical active ROM. Thus, it could be interesting to integrate this online tool in the physiotherapy degree, as well as in physiotherapists’ continuous training [59,60,61] with platforms like Google Forms, the one used in our study [62,63,64]. Video analysis of the cervical ROM could be a part of an effective screening evaluation during pandemics, and with patients who live far away from the health professional or with complications in traveling caused by neck dysfunction or medication [65].

Our study presents the following limitations. As a result of COVID-19 safety precautions, our sample was reduced from 8 asymptomatic subjects [1,9,37,38] to a single asymptomatic subject who simulated different ROMs. Otherwise, the students found it difficult to determine end of movements by the measurement methodology such as video perspective or video sequencing. Those considerations must be considered for further studies. Additionally, our study does not provide the values of validity and reliability in a clinical background and focuses only on the analysis of cervical ROM and not on a full telemedicine evaluation for neck pain including also clinical history, inspection, functional movement patterns, strength and provocative testing [47], so we should be cautious when extrapolating these data clinically.

For further studies we suggest to improve the screening capacity of this telemedicine evaluation, adding other components of the cervical evaluation, trained physiotherapists to the examiners group [41,66] and symptomatic subjects [21,24,27,51,66]. Additionally, regarding ROM clinical evaluation, examiners should be trained to increase their accuracy by describing normal cervical ROM, including schematic images with ROM reference according to different positions in each spatial plane and through successive real videos together with the values of the movement performed according to the CROM.

## 5. Conclusions

The interexaminer reliability and validity of the quantification of active cervical movement during online visual inspection was shown to be good to excellent for flexion-extension and lateral flexions and excellent for rotations.

## Figures and Tables

**Table 1:** Reliability of quantity of motion with online visual estimation vs. CROM, for flexion-extension, lateral flexions and rotations.

Physiotherapy Course	Reliability of “ROM in Visual Inspection”	Reliability of “Classification of Mobility”
FLEXION–EXTENSION		
2nd Course	0.75 (0.30–0.91)	0.03
3rd Course	0.96 (0.88–0.98)	0.62
4th Course	0.81 (0.47–0.98)	0.52
LATERAL FLEXIONS		
2nd Course	0.91 (0.75–0.97)	0.51
3rd Course	0.93 (0.81–0.98)	0.54
4th Course	0.81 (0.45–0.93)	0.26
ROTATIONS		
2nd Course	0.93 (0.80–0.98)	0.90
3rd Course	0.97 (0.90–0.90)	0.58
4th Course	0.96 (0.90–0.99)	0.74

ROM in visual inspection: ICC (lower bound and upper bound of the 95% confidence interval); Classification of mobility: Kappa index. Poor <0.5; Moderate 0.5–0.75; Good 0.75–0.90; Excellent >0.90.

**Table 2:** Validity of online visual estimation vs. CROM for flexion-extension, lateral flexions and rotations.

Physiotherapy Grade	Validity of “ROM in Visual Inspection”
FLEXION–EXTENSION	
2nd Course	0.94 (0.83–0.98)
3rd Course	0.83 (0.50–0.94)
4th Course	0.96 (0.88–0.99)
LATERAL FLEXIONS	
2nd Course	0.88 (0.67–0.96)
3rd Course	0.91 (0.75–0.97)
4th Course	0.90 (0.72–0.97)
ROTATIONS	
2nd Course	0.90 (0.71–0.96)
3rd Course	0.96 (0.88–0.99)
4th Course	0.93 (0.80–0.88)

ROM in visual inspection: ICC (lower bound and upper bound of the 95% confidence interval).

## Data Availability

The data sets analyzed during the current study are available from the corresponding author on reasonable request. All data analyzed during this study are included in this published article.
